# Molecular Phylogenetic Analysis of *Candida krusei*

**DOI:** 10.1007/s11046-022-00640-x

**Published:** 2022-06-11

**Authors:** Marianna Domán, László Makrai, Krisztián Bányai

**Affiliations:** 1 Veterinary Medical Research Institute, P.O. Box 18, Budapest, 1143 Hungary; 2 Department of Microbiology and Infectious Diseases, University of Veterinary Medicine, Budapest, 1143 Hungary; 3 Department of Pharmacology and Toxicology, University of Veterinary Medicine, Budapest, 1078 Hungary

**Keywords:** *Candida krusei*, *Pichia kudriavzevii*, Molecular phylogenetics, Multilocus sequence typing, Haplotype

## Abstract

Revealing the phylogenetic relationships of *Candida krusei* strains (sexual form *Pichia kudriavzevii*) is a prerequisite for understanding the evolution of its virulence-associated mechanisms and ecological lifestyles. Molecular phylogenetic analyses based on entire internal transcribed spacer region (ITS) and multilocus sequence typing (MLST) data were carried out with sequences available in public databases and Hungarian isolates from animals obtained for the study. The ITS haplotype network yielded a high frequency haplotype at the centre of the network (H1; *n* = 204) indicating that various selective pressure might resulted in population expansion from H1. MLST analysis identified three new genotypes among animal-derived isolates, therefore overall 203 sequence types were investigated to determine the population structure of *C. krusei*. The most commonly encountered sequence types were ST 17 and ST 67. Phylogenetic analyses showed diverse genetic construction of *C. krusei* population. Evidence of potential recombination events were also observed that might play some role in high intraspecies genetic variability among strains, however, the limited data of *C. krusei* genotypes from different countries prevented us to identify accurate evolutionary routes of commensal and pathogenic strains or species-specific lineages. Further expansion of *C. krusei* MLST database may promote the better understanding of the mixed evolutionary history of this species.

**Supplementary Information:**

The online version contains supplementary material available at 10.1007/s11046-022-00640-x.

## Introduction

Pathogenic *Candida* species have widely contributed to the increasing morbidity, mortality and excess medical costs of healthcare-associated infections worldwide [1]. *C. albicans* remains the predominant etiological agent of candidiasis, however, the intensive use of some antifungal drugs in human healthcare has promoted a shift in the epidemiology of candidiasis towards non*-albicans Candida* species that are naturally less susceptible to drugs [2]. According to a global surveillance program covering twenty years (1997–2016), the four most frequently isolated non*-albicans Candida* species in descending order include *C. glabrata* (2,860; 18.7%), *C. parapsilosis* (2,433; 15.9%), *C. tropicalis* (1,418; 9.3%), and *C. krusei* (421; 2.8%) [3]. Among these emerging yeast pathogens *C*. *krusei* is the least-well studied despite of its high mortality rate (40–59%) especially in patients with neutropenia and hematologic malignancies [4, 5]. *C. krusei* is a transient commensal of the mucosal membrane of healthy individuals. In veterinary aspect, this organism has also been associated with diseases and deteriorate the health conditions in animals. *C. krusei* is a relevant pathogen involved in mycotic mastitis of cows resulting in reductions of milk yield and quality. Gastrointestinal diseases in birds caused by *C. krusei* have also been reported [6–8].

The emergence of antifungal resistance threatens the effective treatment of fungal infections. Azoles are one of the most common antifungal drugs used for long-term antifungal prophylactic treatment of immunocompromised individuals and applied in agricultural practices. *C. krusei* is a potential multidrug-resistant yeast due to its intrinsic resistance to fluconazole (with more than 96% of human clinical and veterinary isolates being fluconazole-resistant) as well as its acquired resistance developed to some other antifungal drugs (amphotericin B, echinocandins) [6, 9–11].

Additional specific characteristics of this species is the presence of sexual reproduction. The sexual form (teleomorph) of *C. krusei* is *Pichia kudriavzevii* that was proposed by Kurtzman and colleagues in 1980 [12]. *P*. *kudriavzevii* isolates are widely distributed in nature and due to its stress tolerance it has an increasing role in biotechnology mainly for production of bioethanol [13]. Moreover, some strains are candidate probiotics [14]. Recent whole genome sequencing supported that there is no genetic distinction between the two species [15]. Phylogenetic analysis of ribosomal DNA (rDNA) and other conserved genes along with genome sequences revealed large evolutionary distance between *C. krusei* and other medically relevant *Candida* species that further supported the re-classification of this organism in the *Pichia* genus [15, 16]. In accordance with the guidelines to avoid multiple denomination of any fungus species, the general usage of the name *P. kudriavzevii* (sexual form) has been suggested but has not yet been enacted; therefore the more conventional name, *C. krusei*, will be used in our article.

Relatively little genetic or genomic investigation has been carried out on *C*. *krusei* isolates so far. Sequence data from multiple loci facilitates determinations of population structures and epidemiological correlates of properties, such as geographical and anatomical origins of isolates and their transmission within and between hosts, therefore multilocus sequence typing (MLST) has gained widespread acceptance as a tool for exploring strain-level differences within microbial species [17–20]. Another typing scheme based on short tandem repeats for *C. krusei* have recently been developed that may facilitate the follow up of outbreaks in healthcare settings in the future [21]. A previous MLST study of *C. krusei* indicated that isolates have diploid, heterozygous genome [18], but phylogenetic analysis using the extended data deposited in MLST database to determine the genetic diversity and population structure of strains has not performed since 2007. The content of the MLST database has expanded since this original report on the genetic structure of *C. krusei*; therefore the authors felt that new analyses would permit a deeper understanding of the genetics and evolutionary history of this important opportunistic pathogen.

## Materials and Methods

### Sample Collection and Identification of Species

Eight *C. krusei* isolate were cultured from swab samples sent for species identification to our laboratory. All samples were obtained from the uterus of different cows. One isolate from our culture collection was also included in the study, which originated from the oesophagus of a duck diagnosed with gastrointestinal mycosis. Swab samples were inoculated on Sabouraud dextrose agar supplemented with chloramphenicol and incubated at 35 °C for 48 h. The cultures were identified by Matrix-assisted laser desorption/ionization time of flight mass spectrometry (MALDI-TOF MS) and molecular method by sequencing of internal transcribed spacer (ITS) region of fungal rDNA [22].

After culturing isolates for two days, the genomic DNA was extracted and purified directly from a single colony of each isolate by using the Fungi/yeast genomic DNA extraction kit (Favorgen, Ping-Tung, Taiwan). DNA samples were stored at − 20 °C until analysis. The complete ITS region, including ITS1 and ITS2 and the 5.8S region of rDNA were amplified with fungus-specific universal primers ITS1 (5′-TCCGTAGGTGAACCTGCGG-3′) and ITS4 (5′-TCCTCCGCTTATTGATATGC-3′). The reaction was performed in a final volume of 15 μl including 1 µl fungal DNA, 2 µl 10 × DreamTaq buffer, 0.5 µl dNTP (10 mM), 0.5 µl forward and reverse primers (10 µM each), 0.1 µl DreamTaq DNA polymerase (5 U/*µ*l; Thermo Fisher Scientific, Waltham, MA, USA) and 10.4 µl distilled water. The reaction conditions were as follows: initial denaturation step at 95 °C for 3 min, 40 cycles of 95 °C for 30 s, annealing at 50 °C for 30 s, extension at 72 °C for 1 min, and a final extension step at 72 °C for 10 min. Both strands of purified gene fragments were sequenced using ITS1 and ITS4 primers with BigDye Terminator v3.1. cycle sequencing kit (Thermo Fisher Scientific, Waltham, MA, USA) on an ABI Prism 3130 Genetic Analyzer (Applied Biosystems). Sequences were edited and assembled using MEGA v6 software [23] and compared with sequences in database by the BLAST sequence analysis tool (https://blast.ncbi.nlm.nih.gov/Blast.cgi).

### Multilocus Sequence Typing of *C. krusei* Isolates

The MLST scheme employed for *C. krusei* genotyping was based on partial amplification and sequencing of six protein-coding genes (*HIS3*, *LEU2*, *NMT1*, *TRP1*, *ADE2*, and *LYS2D*) according to the international reference MLST scheme established for this species. Six independent PCR amplifications were performed for each isolate. The primer sets and their amplicon lengths were described in detail elsewhere [18]. Experimental conditions used in PCRs and Sanger sequencing were detailed above.

Heterozygosity was identified by the presence of two coincident peaks at the same polymorphic loci on the forward and reverse sequence chromatograms and the consensus sequences of six loci of all isolates were defined [24]. Each nucleotide sequence was compared to those in the MLST database (https://pubmlst.org/organisms/candida-krusei) [25]. Sequence variation in each locus was assigned an allele number. Those sequences that did not match with any of the preexisting sequences was given a new allele number by the curator. The combination of the six allele numbers defined a unique sequence type (ST) of an isolate.

### Phylogenetic and Population Structure Analysis

To assess the intraspecific variability of the ITS region of *C. krusei* isolates as a universal fungal barcode, we selected sequences deposited in GenBank for phylogenetic analysis. Sequences that were not long enough (< 470 bp) were eliminated. Eight ITS sequence were excluded from the analysis as they were more closely related to *Candida pseudolambica*, *Pichia bruneiensis* and *Pichia fermentans* than to *C. krusei* (MN710485-MN710491, MT781361). Neighbor-joining (NJ) tree was constructed from 268 ITS sequences using JC + G substitution model with bootstrap of 1,000 replications. As NJ tree showed minimal differences between sequences, phylogenetic network was also generalized that better model the phylogenetic relations between isolates by creating haplotypes from diploid sequence data and considering the possibility of recombination, hybridization, gene conversion and gene transfer. Sequence polymorphism analysis was carried out with DnaSP version 6 software and haplotype network file was created [26]. Haplotypes were defined as unique combination of SNPs along a particular sequence. Haplotype diversity values range from 0 to 1, with those closer to 1 indicating higher variability. Haplotype network was generated by median-joining method [27] implemented in NETWORK v4.6.1.0 (Fluxus Technology, Suffolk, UK).

For multilocus sequence analysis, the sequenced loci were concatenated into a single sequence and single nucleotide polymorphisms (SNPs) were converted as described by Tavanti et al. (2005) [28] to label homozygous and heterozygous sites in order to allow the cluster analysis of diploid sequence data. Briefly, each base at the polymorphic sites rewritten twice for a homozygous (A, C, G, or T) datum or once each for the two component bases for a heterozygous (K, M, R, S, W, or Y) datum. All sequences available in the MLST database were involved in phylogenetic analysis. The genetic relatedness between the investigated strains was evaluated by unweighted pair group method with arithmetic averages (UPGMA) algorithm with p-distance model in MEGA v6 software. Random bootstrapping of 1,000 operations was used for the construction. Clonal complexes (CCs) of isolates that differed in sequence at only one of the six loci (single-locus variant analysis) were predicted by the goeBURST algorithm (http://www.phyloviz.net/goeburst/) to investigate the evolutionary relationships between isolates. CC is a set of STs that are all believed to be descended from the same founding genotype. STs that could not be assigned to any group were called singletons. Haplotype diversity was calculated and possible recombination events were estimated using the DnaSP v6 software [26, 29].

## Results

**Fig. 1:**
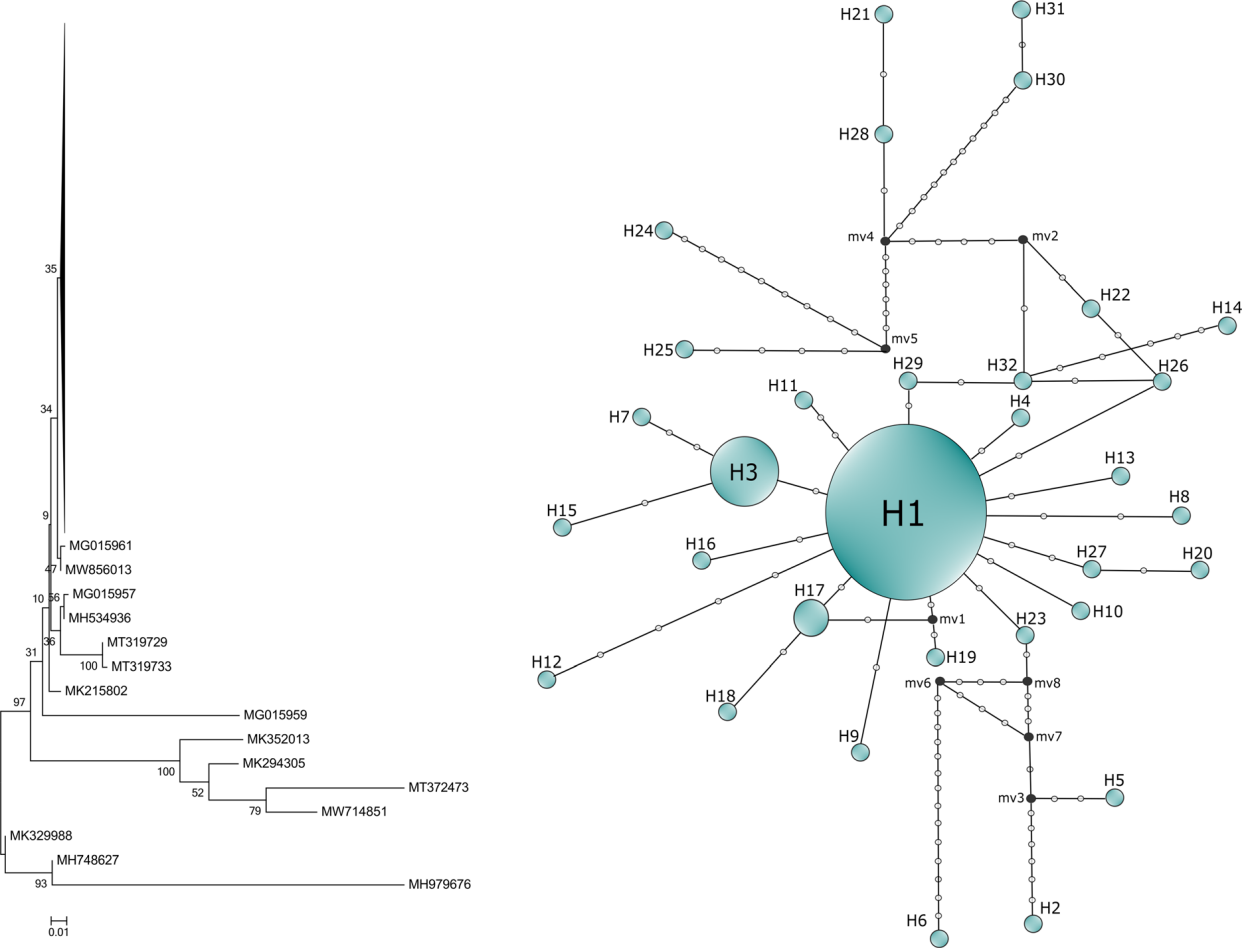
Phylogenetic tree of *Candida krusei* ITS sequences and network analysis of ITS haplotypes by median-joining method. The compressed branch of the tree contains 253 sequences. Numbers along the branches indicate bootstrap values. The size of the circumference is proportional to the haplotype frequency in the network. Mutational steps are represented by white dots. The black dots (median vectors) represent unsampled or extinct haplotypes in the population

Based on aligned ITS sequences, 76 variable sites and 32 haplotypes with haplotype diversity of 0.3869 were determined. Low intraspecific diversity was found among isolates. Most of the sequences were identical and belonged to 3 haplotypes (H1, H3 and H17). Our samples also clustered to the H1 group along with reference strain ATCC 6258. The ITS haplotype network yielded a high frequency haplotype at the centre of the network (H1; *n* = 204), followed by H3 (*n* = 26), H17 (*n* = 4) and other haplotypes with a single sequence indicating that various selective pressure might resulted in population expansion from H1. Several haplotypes differed in only one nucleotide from each other which is evident in network analysis but not in NJ phylogenetic reconstruction (Fig. 1).

**Fig. 2:**
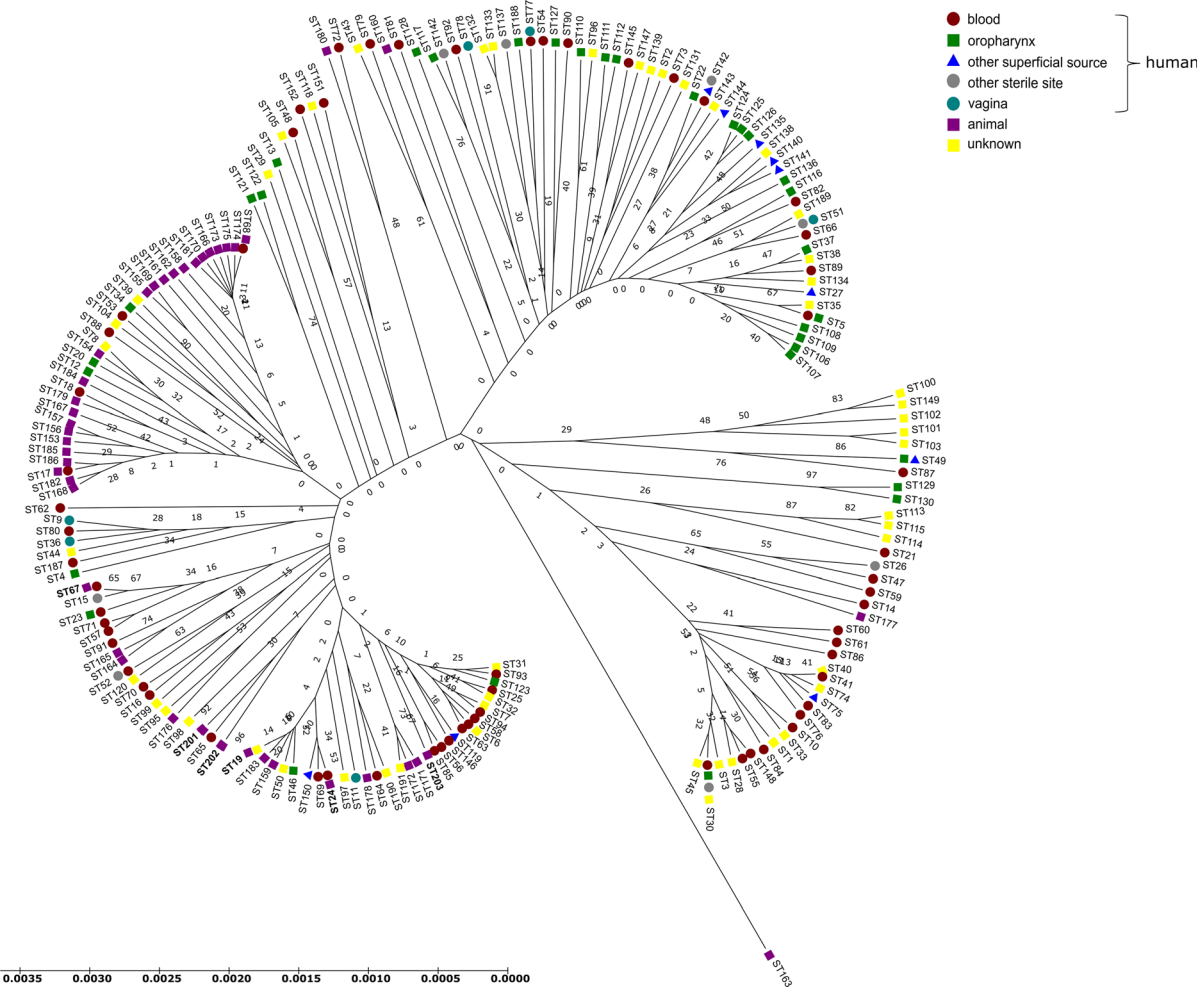
Phylogram constructed by unweighted pair group method with arithmetic mean analysis (UPGMA) of *Candida krusei* sequence types. The scale bar indicates P-distance. The arbitrarily selected P-distance cutoff was 0.0028. Hungarian isolates are highlighted with bold numbers. Numbers along the branches indicate bootstrap values

**Fig. 3:**
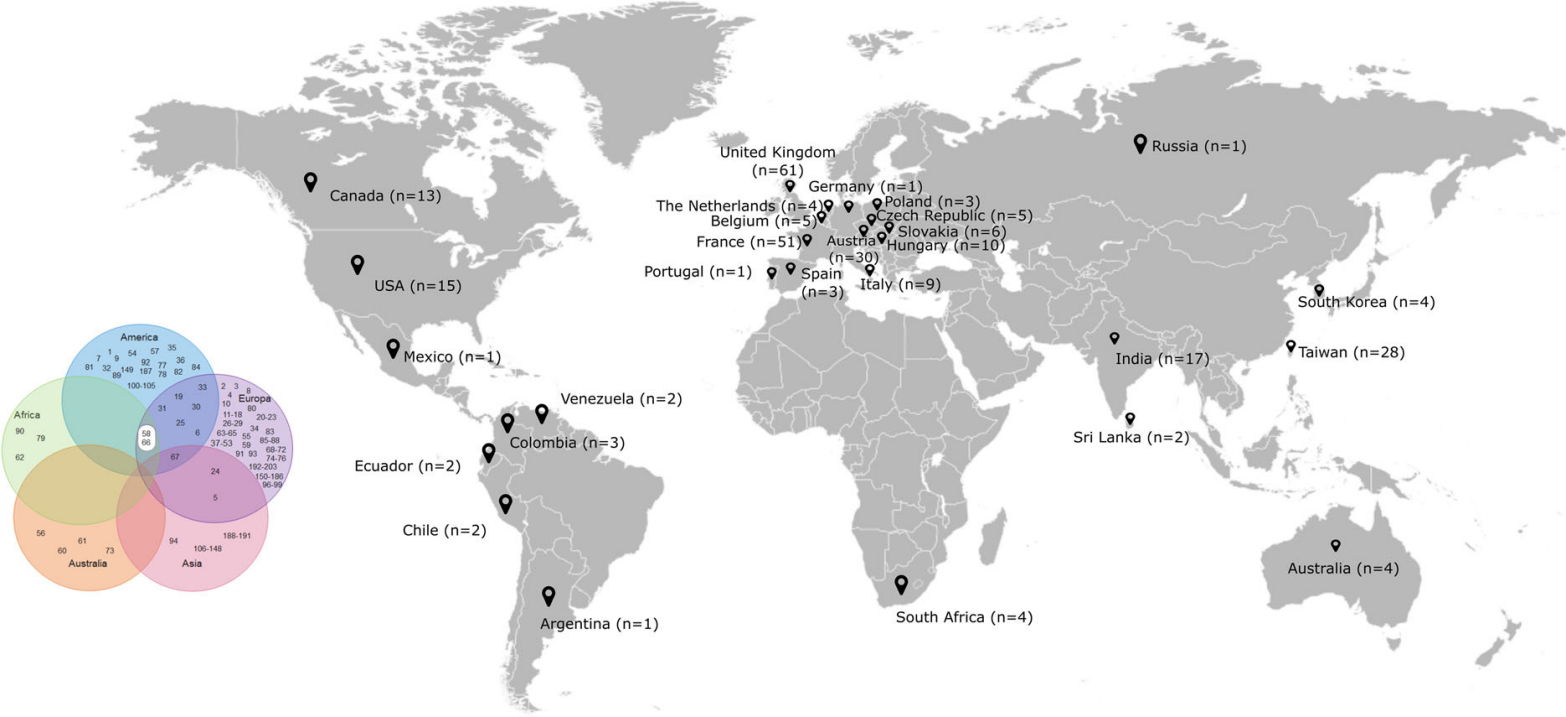
Number of isolates submitted to MLST database from different countries and distribution of sequence types among continents. ST numbers highlighted with white background are occurred in only Africa and Europe

Concatenated sequence data from the six partial genes yielded a sequence of 2,847 bp for each isolate whose sequences were determined in this study. In two animal-derived isolates a shared new allele was identified in *LEU2*. Four isolates were assigned to previously described STs whereas another five isolates were classified in new MLST genotypes (ST numbers: 201–203) (Table 1). Together with our newly submitted data, a total of 203 STs available in the *C. krusei* MLST database were investigated in the study. Overall, 76 polymorphic sites were found among all examined loci. The *NMT1* locus produced the highest number of polymorphic sites (*n* = 35), while *HIS3* displayed the lowest (*n* = 23). The most commonly encountered strain types were ST 17 (13 isolates) and ST 67 (18 isolates). These isolates were obtained from human blood or an animal source and the geographical origins of isolation were Austria, Canada, Czech Republic, France, Hungary, Italy, Taiwan, United Kingdom, and Venezuela. Other STs were represented by less than 10 isolates. In order to reveal the phylogenetic relationship between isolates, we performed cluster analysis of STs submitted to the MLST database by the UPGMA method. *C. krusei* STs could be subgrouped into five clusters with an approach described by Jacobsen et al. (2007) [18], however, the bootstrap values for the nodes in the dendrogram were extremely low hampering the accurate distinction of internal structure. The largest group was subtype 1 containing 52.06% of STs, followed by subtype 2 (28.35%), subtype 4 (15.46%), subtype 3 (3.61%) and subtype 5 (0.51%) (Fig. 2). STs from human blood and animal source were the main components of group subtype 1, although, isolates from animals were submitted only from two countries (France and Hungary). Subtype 2 predominantly consisted of STs from the oropharynx (including sputum and bronchoalveolar lavage) of human patients. In contrast to other members of the group subtype 3, the anatomical origin of ST 49 and ST 87 was known (oropharynx/other superficial source and blood, respectively), while subtype 4 was prevailingly composed of blood isolates. One ST obtained from an animal formed group subtype 5 alone. In general, several STs were identified in all subtypes whose origin was unknown (Table 2, Fig. 2). Unfortunately, information on STs from different geographical origin is incomplete and thus, the correlation between subtypes and host specificity or anatomical source could not be determined (Fig. 3).

**Table 1:** Origin of *Candida krusei* isolates and MLST genotypes obtained in the study

Host	Isolate number	Origin	MLST loci						ST	CC
			*HIS3*	*LEU2*	*NMT1*	*TRP1*	*ADE2*	*LYS2D*		
Cow	DA27	Uterus	1*	14	8	5	5	10	201	S
Cow	DA29	Uterus	4	9	8	8	2	9	67	7
Cow	413	Uterus	5	27	4	5	3	10	202	S
Cow	414	Uterus	5	27	4	5	3	10	202	S
Cow	766	Uterus	1	2	3	5	5	10	19	4
Cow	775	Uterus	1	2	3	5	5	7	24	4
Cow	802	Uterus	1	14	8	5	5	10	201	S
Cow	803	Uterus	4	9	11	5	5	4	203	S
Duck	Om-10	Oesophagus	1	2	3	5	5	10	19	4

*ST* Sequence type assigned by *C. krusei* MLST database based on allelic profiles (sequence number combinations) * numbers are designated based on the composite sequence of allele variations; *CC* Clonal Complex determined by eBURST; *S* singleton

**Table 2:** Subtype distribution of *Candida krusei* isolates from different anatomical source

Subtype	No. (%) of STs from blood	No. (%) of STs from oropharynx	No. (%) of STs from other superficial source	No. (%) of STs from other sterile site	No. (%) of STs from vagina	No. of (%) STs from animal
1	33 (32.67)	10 (9.90)	2 (1.98)	2 (1.98)	3 (2.97)	39 (38.61)
2	13 (23.63)	19 (34.54)	6 (10.90)	4 (7.27)	3 (5.45)	1 (1.81)
3	15 (50)	3 (10)	2 (6.6)	2 (6.6)	–	1 (3.3)
4	1 (14.28)	1 (14.28)	1 (14.28)	–	–	–
5	–	–	–	–	–	1 (100)

**Fig. 4:**
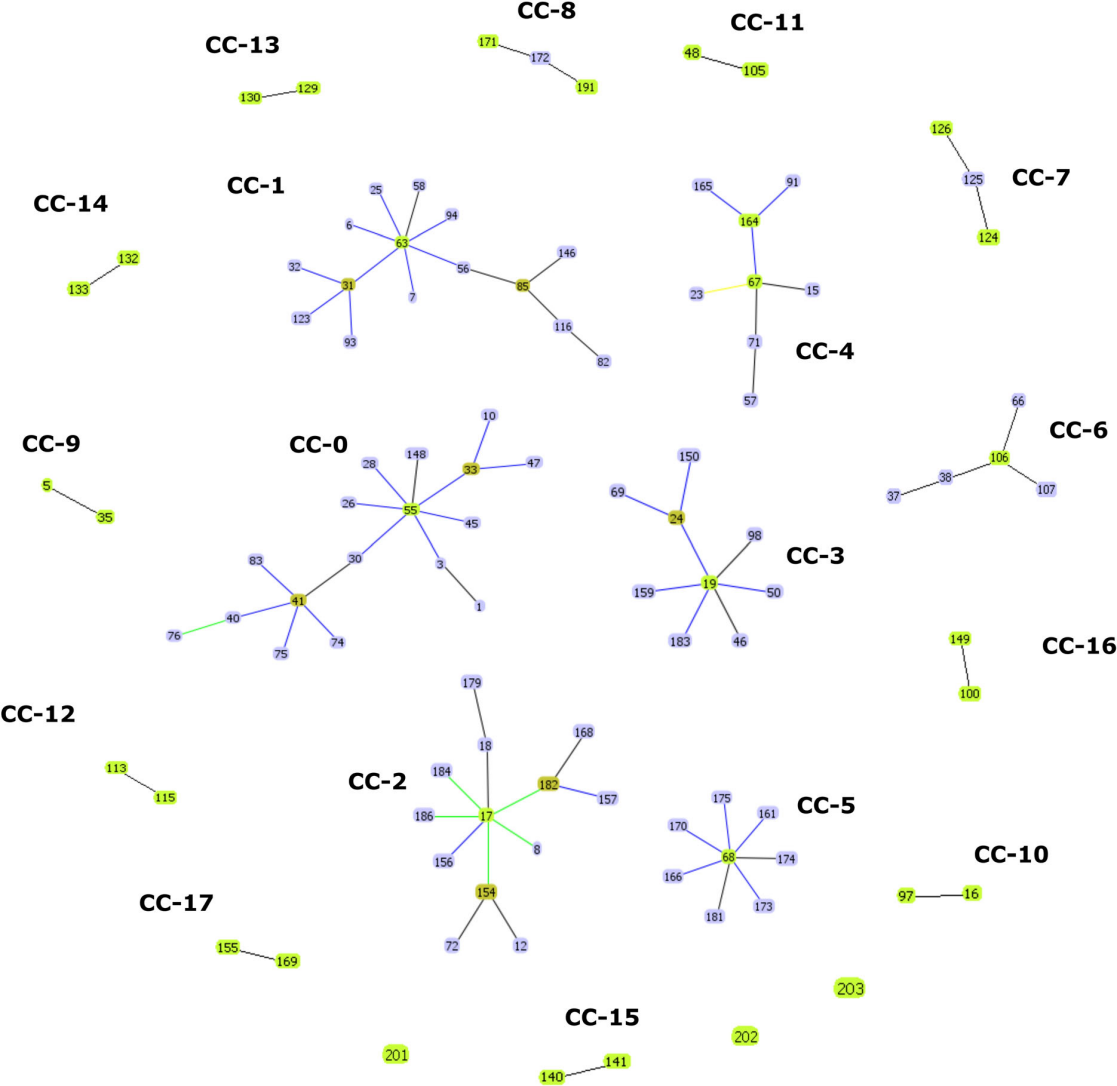
eBURST snapshot for *Candida krusei* sequence types available in the MLST database. The illustration shows clonal complexes (CCs). Only Hungarian singletons (i.e. ST that could not be assigned to any group) were marked

The goeBURST algorithm was used to identify putative ancestral STs and their closest relations. Evaluation of genotypic relationship of strains resulted 18 CCs and 94 singletons. CCs generated by goeBURST were numbered starting from 0 (for the CC with most STs). Our isolates with known STs clustered to CC-3 (ST 19 and ST 24) and CC-4 (ST 67), while newly identified STs were singletons. The ST 24 was putatively evolved from the ST 19 group founder due to loss of heterozygosity considering nucleotide positions 2,467 and 2,659 in *LYS2D*. In CC-4, ST 67 or ST 164 was predicted as founder of the group. Interestingly, ST 67 was the most prevalent genotype that was isolated globally (including Asia, Europe, North America and South America). Besides Hungarian isolates, *C. krusei* MLST data from animals were reported only from France. Of note, ST 17, ST 19, ST 24 and ST 67 were detected from animal samples in both countries. Moreover, ST 17 and ST 67 were the most prevalent genotypes identified even from humans and animals worldwide, raising the question whether strains that belong to these genotypes are better adapted to colonise or infect different hosts. Overall, ST 17, ST 19, ST 24, ST 67 and ST 68 were detected from both humans and animals and these genotypes were mostly assigned as probable ancestors of CCs (Fig. 4). To provide a more accurate representation of the relationships among genetic groups, haplotype analysis of each six coding regions and the concatenated sequences were carried out and recombination events were estimated, which revealed evidence of potential recombination among the loci assessed suggesting the mixed ancestry of strains (Table 3).

**Table 3:** Haplotype diversity of MLST genes and estimated recombination events regarding 203 sequence types (STs) of *Candida krusei*

MLST genes	Number of haplotypes (Haplotype diversity)	Variable sites	Minimum number of putative recombination events
*ADE2*	13 (0.8937)	10	1
*HIS3*	14 (0.8899)	12	1
*LEU2*	15 (0.9078)	10	3
*LYS2D*	18 (0.9085)	10	3
*NMT1*	21 (0.9151)	17	2
*TRP1*	21 (0.9175)	17	3
Concatenated sequences	223 (0.9919)	76	20

## Discussion

Conserved and variable rDNA regions are suitable and universally applied for comparative analysis to identify organisms at taxonomic levels and clarify phylogenetic relationships between species and populations [30]. In recent years, many studies have been denoting some limitations in using ITS for delimiting fungal species such as the lack of differentiation of closely related species and the presence of non-homologous copies of ITS in the genome [22]. Intragenomic ITS variations that was observed in *Candida* and *Pichia* species as well may derive from recombination, gene duplication and point mutations produced by DNA replication errors [22, 31, 32]. However, despite these limitations, the utilization of the rDNA ITS region for sequence analysis still appears to be the most reliable method for the rapid and accurate molecular identification of fungal pathogens. Furthermore, phylogenetic, taxonomic and population dynamics studies are substantially concerned with polymorphisms in the ITS region and are proven to be particularly useful for the delineation of ascomycetous yeasts [30, 33]. Comparative analysis of *C. krusei* ITS sequences including those deposited in public genomic databases showed low intraspecies variability as the majority of sequences belong to H1 haplotype, while the remaining 31 haplotypes are represented by relatively few sequences. The sampling efficiency for network analysis has been supported by the low number of median vectors which predicts the existence of unsampled or extinct haplotypes. Nevertheless, several haplotypes could be evolved from H1 with numerous mutational steps suggesting higher variability of ITS sequence of *C. krusei* than formerly noted [33].

The *C. krusei* strains were also studied by MLST analysis. MLST provides relevant information for population genetic studies and for epidemiologic investigations with higher level of reproducibility and minimal subjectivity in analysis compared with other technologies [24]. The genes chosen to be sequenced for MLST conventionally encoded metabolic functions that are subject to stabilizing selection, because diversifying selection may obscure relationships among isolates [24, 34]. *C. krusei* STs in MLST database (*n* = 203) lag far behind the number of STs of other *Candida* species with diploid genome that can be genotyped by the MLST approach (*C. albicans*, *n* = 3,669; *C. tropicalis*, *n* = 1,245). Jacobsen et al. [18] devised a MLST scheme ideal for strain typing of *C. krusei* using 129 human isolates and distinguished 94 STs. Fourteen years later, we performed an extended phylogenetic analyses with 302 isolates and 203 STs from the *C. krusei* MLST database. As a result of this timely update we have gained more comprehensive insight into the evolutionary history of *C. krusei* using multiple analyses by inclusion of strains from different source. According to our results, similar to the previous observation, STs can be subtyped. Furthermore, the majority of strains originated from animals belonged to Subtype 1. Although certain genotypes were determined from one host (e.g. ST 153–186 originated from animals) or appeared in one continent (e.g. ST 56, ST 60, ST 61 and ST 73 were noted exclusively from Australia), no unequivocal evidence of geographical associations of STs were seen due to the comparatively low numbers of isolates typed so far. This is consistent with findings that this species is the least frequently isolated among the five most common clinically relevant *Candida* species and typing of strains obtained from animals and environmental sources are somewhat neglected. In addition, submitting genotyped strains to MLST database requires anatomical source of isolation. Thus, strains from other sources (e.g. milk, cheese) [35] marked as unknown prevents a better resolution of phylogenetic relations. Most of the STs that originated from animals (eg., ST 19, ST 67) were putative group founders or differed only by a single allele when using cluster analysis (eg., ST 24, ST 182, ST 184, ST 186) suggesting that these strains were less exposed to changing environmental conditions. The high rate of singletons found in eBURST analysis and the evidence of recombination, which might be an explanation of lower bootstrap values in the UPGMA dendrogram, further supports the unexclusively clonal reproduction of *C. krusei,* a finding that coincided with previously published data [15, 18].

In conclusion, studies on population structure of *C. krusei* are currently incomplete. Although recombination events may play some role in the observed high intraspecies genetic variability, the limited data of *C. krusei* genotypes from different countries submitted to MSLT database make it difficult to reveal accurate evolutionary routes of commensal and pathogenic strains or species-specific lineages. Some genotypes were identified from humans and animals that may indicate certain genotypes are better adapted to diverse environmental niches. A significant increase of sequence data together with isolate-associated metadata in the MLST database will be needed for a more robust resolution of the population structure of *C. krusei*.

## Supplementary Information

Below is the link to the electronic supplementary material.

Supplementary file 1 (FASTA 554 kb)

## Data Availability

Generated ITS sequences were deposited in GenBank under the following accession numbers: OL470260-OL470268. Allele sequences and concatenated sequences of the six genes used for MLST are available at: https://pubmlst.org/organisms/candida-krusei. The converted concatenated dataset of loci used for cluster analysis can be found in Supplementary file 1.
